# Complete Genome Sequence of Staphylococcus aureus Strain 834, Isolated from a Septic Patient in Japan

**DOI:** 10.1128/MRA.01477-20

**Published:** 2021-03-04

**Authors:** Hisaya K. Ono, Yasunori Suzuki, Hiroaki Kubota, Krisana Asano, Shinji Takai, Akio Nakane, Dong-Liang Hu

**Affiliations:** a Laboratory of Zoonoses, Kitasato University School of Veterinary Medicine, Towada, Japan; b Department of Microbiology and Immunology, Hirosaki University Graduate School of Medicine, Hirosaki, Japan; c Laboratory of Animal Hygiene, Kitasato University School of Veterinary Medicine, Towada, Japan; d Department of Microbiology, Tokyo Metropolitan Institute of Public Health, Tokyo, Japan; University of Arizona

## Abstract

Here, we report the complete genome sequence of Staphylococcus aureus strain 834, which was isolated from a septic patient in Japan and showed strong virulence and methicillin resistance. The complete genome consists of a 2,838,668-bp chromosome and a 24,653-bp plasmid. Genome annotation predicts 2,670 coding sequences, 16 rRNAs, and 61 tRNAs.

## ANNOUNCEMENT

Methicillin-resistant Staphylococcus aureus (MRSA) exhibits resistance to multiple antimicrobials, is a leading cause of nosocomial infections, and often causes life-threatening diseases (sepsis, deep organ abscesses, and toxic shock syndrome). Here, we report the complete genome sequence of strain 834, which was isolated from a septic patient at Hokkaido University Hospital in Sapporo, Japan (1). According to our previous studies, S. aureus strain 834 has strong virulence (1–4). In a mouse model of systemic infection, no mouse survived after challenge with a lethal dose of bacteria (5 × 10^7^ CFU) (2–4).

Strain 834, which had been stored at −80°C in tryptic soy broth supplemented with 10% glycerol, was grown in brain heart infusion broth at 37°C with shaking. Genomic DNA was extracted from the cultured cells, after treatment with lysostaphin (Wako Pure Chemicals, Osaka, Japan) and RNase A (400 μg/sample; Nippon Gene Co., Ltd., Toyama, Japan), using a DNeasy blood and tissue kit (Qiagen GmbH, Hilden, Germany). The concentration and purity of the extracted DNA were determined using a Qubit double-stranded DNA (dsDNA) high-sensitivity (HS) assay kit (Thermo Fisher Scientific, Waltham, MA, USA) and a NanoDrop 2000c spectrophotometer (Thermo Fisher Scientific), respectively. This DNA sample was analyzed by both short-read sequencing and long-read sequencing. A paired-end sequencing library was prepared using a Nextera XT DNA sample preparation kit (Illumina, Inc., San Diego, CA) according to the manufacturer’s instructions. Illumina paired-end (2 × 300-bp) reads were obtained using a MiSeq system (Illumina). MiSeq raw reads were trimmed using Trim Galore v0.4.3 (https://www.bioinformatics.babraham.ac.uk/projects/trim_galore) with default settings. Long-read sequence data were obtained with a MinION system (Oxford Nanopore Technologies, Oxford, UK). A DNA library was prepared using a ligation sequencing kit (SQK-LSK109) and native barcoding expansion (NB09 of EXP-NBD104), and the prepared library was subsequently loaded into a MinION flow cell (R9.4). The MinION sequencing run was performed over 48 h using MinKNOW v19.12.5. Base calling and Fastq barcoding were performed using Guppy v2.3.7. MinION raw reads were trimmed with NanoFilt v2.6.0 (5) at a quality threshold of 10. Hybrid assembly of the MiSeq and MinION reads was performed using Unicycler v0.4.2 (6). Genome error correction, circularization, and rotation were implemented in the Unicycler pipeline. The complete genome sequence was annotated using DFAST v1.2.6 with the default parameters (https://dfast.nig.ac.jp) (7, 8). Assembly statistics, general genome information, and relevant characteristics are summarized in Table 1.

**TABLE 1:** Assembly statistics, general genome information, and relevant characteristics of the Staphylococcus aureus strain 834

Strain and genome information	Data for Staphylococcus aureus strain 834
Strain information	
Origin	Septic patient at Hokkaido University Hospital (Sapporo, Japan)
Yr of isolation	1985
Sequence type	ST5
Assembly and genome statistics	
MinION sequencing	
No. of reads	156,090
Total no. of bases	746,651,550
After trimming with NanoFilt	
No. of reads	91,869
Read length *N*_50_ (bp)	9,979
Total no. of bases	457,925,254
MiSeq sequencing	
No. of reads	907,262
Total no. of bases	163,219,976
After trimming with Trim Galore	
No. of reads	907,262
Total no. of bases	162,023,447
Coverage (×)	217
Chromosome features^*a*^	
Genome size (bp)	2,838,668
G+C content (%)	32.9
No. of CDSs^*b*^	2,670
Coding proportion (%)	83.7
No. of rRNAs	16
No. of tRNAs	61
No. of CRISPR regions	0
p834 features^*a*^	
Genome size (bp)	24,653
G+C content (%)	28.7
No. of CDSs	29
Coding proportion (%)	75.4
No. of rRNAs	0
No. of tRNAs	0
No. of CRISPR regions	0

a All genomic statistics are output from the DFAST pipeline.

b CDSs, coding sequences.

The chromosome of strain 834 was 2,838,668 bp (G+C content, 32.9%). The numbers of predicted coding sequences, rRNAs, and tRNAs in the genome were 2,670, 16, and 61, respectively. Compared with the closely related strain N315 (sequence type 5 [ST5]), strain 834 had a putative intact prophage (ϕSa834) at positions 1515024 to 1576239; 67 phage hit proteins and 7 hypothetical proteins existed on the prophage (9). Strain 834 possessed one plasmid, p834. The plasmid is completely identical to pN315, which is harbored by S. aureus strain N315.

Consequently, strain 834 had a genetic background similar to that of N315, but there was an insertion, a putative intact prophage. The difference of prophage insertion may contribute to the pathogenesis.

### Data availability.

This whole-genome project has been deposited in DDBJ/ENA/GenBank under accession numbers AP024170 and AP024171 for the chromosome and plasmid (p834), respectively, BioProject number PRJDB10786, and BioSample number SAMD00258402. The raw data have been deposited in the DDBJ Sequence Read Archive (DRA) under the accession number DRA011194; the accession number for the Illumina sequence data is DRR258631, and that for the Nanopore sequence data is DRR258632.
